# Bibliographical

**Published:** 1885-12

**Authors:** 


### Bibliographical.

Dental Bibliography, a standard reference list of books on dentistry published throughout the world from 1536 to 1585, arranged chronologically and supplemented with a complete cross reference to authors, compiled by G. Crowley, 180 pages, 12°. Philadelphia. S. S. White Dental Manufacturing Co. 1885.

The handy volume under review is the latest addition to the series of special bibliographies which during the last few years have shown the improved views of practical men. The inventor of to-day no longer endeavors by the light of nature alone to improve his art, but wisely goes through all the literature of the subject to see what has been done before him. For this purpose he requires special assistance, such as is given in May's Bibliography of Electricity and other works of the same class.

The only separate publication on dental literature in the English language that had previously appeared is the excellent but confessedly incomplete list of Mr. Oakley Coles, published as a supplement to his address on Dental Literature before the Odontological Society of Great Britain in 1882.

Partial lists had appeared previously in 1793, *Neues Magasin fur Aertze*; 1814, *Dictionnaire de Sciences Medicales.* In the article Dentition, by Fournier, there is a most amusing catalogue in chronological order in which each book is passed in summary review. "Hunter on the Teeth is full of new and useful things. Du Chemant, his book made a sensation, but is forgotten. Fox is remarkable for his excellent principles. Laforgue has shown research and may be consulted with advantage in spite of his extreme ideas and prolixity of style, his chapter on diagnosis shows a want of method and of medical knowledge." Of the same author's dissertation on first dentition, the trenchant critic remarks that what good this pamphlet contains is not new, the rest is paradoxical and above all not medical. Of Galette, he says that his bibliography is long but incorrect, copied mainly from Plouquēt. Fitch in 1829 and Linderer and Carabelli in 1831, give lists of dental works, and Oudot, in the articles Dents and Odontalgie, in the *Dictionnaire de Medecine,* Paris, 1835, gives classified catalogues under the seven following heads: First, second, and third dentitions, changes by age, anomalies, comparative anatomy, and pathology. This writer gives many of the references to the teeth which have appeared in the Greek and Latin authors of the classic and middle ages supplementing the work of Duval. "Advice of the ancient poets on the teeth" which had appeared in 1805.

In 1833 F. Maury and in 1843 Desirabode published extensive bibliographies, the latter being unfortunately separated from the translation published in the Library of Dental Science and inserted at a later date in the pages of the *American Journal of Dental Science.*

In 1844 Goddard added a list of authorities to his Treatise on the Teeth. Buchting, of Nordhausen, published in 1867 Biblotheca Odontiatrica, a bookseller's catalogue of German books and periodicals that had appeared between 1847 and 1866. In 1869 appeared a list in the *Nouveau Dictionnaire de Medecine et de Chirurgie,* in 1882 we have the admirable report of Mr. Coles with its appendix, and in 1883 Gracklauer, of Leipzig, issued a list of German dental literature from 1857 to 1882.

From this rapid glance at what has been attempted in this field, it is clear that in the words of the preface "no attempt to issue a complete and exhaustive dental bibliography has heretofore been made."

In the list of 752 German works, extending from 1536 to the present, it is interesting to note that although the first three named are in the vernacular, the next 166, covering a period of 177 years, are in Latin, with three exceptions, and consist mainly of these presented at the different German universities.

Between 1595 and 1597 there was a very pretty quarrel between Martin, Ruland, Jacob, Horst, and John Ingolstett as to the veracity of the history of a boy who had lately cut a golden tooth in Silesia, the rumor of which spread over Europe to Lyons, where Jacob Frank published the marvellous history. In this connection we would point out that the date assigned to number 75 is probably wrong by a hundred years.

We are glad to see that the name of Urbain Hemard, the author of the first of the 709 French books, is correctly spelled; in all previous lists the authors or printers seem to have used every form but the correct one.

In the English section are 229 books, of which the first dates from 1687, and in the American 308, dating from 1801.

Of the total of 2,047, the indulgent estimate of Mr. Coles might lead us to admit that about 500 are of enduring value.

In a future edition we would suggest a more full description of the books with respect to the number of pages, illustrations, etc.

This work is a most valuable one, and should, as we hope it soon will be, in the library of every dentist in the country, especially of those who have any interest in the literature of our profession. The book is well gotten up, and is an additional witness to the throughness with which the publishers do all their work. Price, $2.00 interleaved copies, $2.50.

